# Co-designing Nova Sleepcare: identifying priority areas for implementation

**DOI:** 10.3389/fped.2026.1760214

**Published:** 2026-07-01

**Authors:** Ishnoor K. Nahal, Myka Estes, Genevieve Currie, Callum Heathcote, Greta Heathcote, Elizabeth Deliscar, Suzanne Deliscar, Megan Thomas, Anamaria Richardson, Deborah Dewey, Sarah J. MacEachern

**Affiliations:** 1 Department of Pediatrics, Cumming School of Medicine, University of Calgary, Calgary AB, Canada; 2 Alberta Children’s Hospital Research Institute, Cumming School of Medicine, University of Calgary, Calgary, AB, Canada; 3 School of Nursing and Midwifery, Mount Royal University, Calgary, AB, Canada; 4 Department of Pediatrics, Dalhousie University, Halifax, NS, Canada; 5 IWK Health, Halifax, NS, Canada; 6 Faculty of Health and Medicine, Lancaster University, Lancaster, United Kingdom; 7 Department of Pediatrics, Faculty of Medicine, University of British Columbia, Vancouver, BC, Canada; 8 BC Children's Hospital Research Institute, Vancouver, BC, Canada; 9 Department of Community Health Sciences, Cumming School of Medicine, University of Calgary, Calgary, AB, Canada; 10 Hotchkiss Brain Institute, University of Calgary, Calgary, AB, Canada

**Keywords:** co-design, implementation, neurodevelopemental disorders, pediatric, sleep disturbance, behaviors of concern

## Abstract

**Background:**

Sleep problems are common in youth with neurodevelopmental disorders and directly contribute to behaviors of concern. While behavioral sleep education improves both issues, a disconnect exists between research and clinical reality: evidence-based sleep interventions are mainly delivered through specialized clinics and multi-week programs, which are often inaccessible to families needing immediate support. To bridge this gap we co-designed with end-users, a physician-delivered, point-of-care active sleep education intervention called Nova Sleepcare that can be directly implemented in clinical and household setting.

**Objective:**

To identify end-user priorities for learning about, implementing, and supporting Nova Sleepcare in clinical and household settings through consensus methods.

**Methods:**

Fifteen end-users (four youth, six caregivers, and five pediatric physicians) participated in structured nominal group technique sessions. Each group addressed questions about: (1) youth and caregiver's preferred methods for learning about Nova Sleepcare from physicians; (2) implementation barriers and support needs at home reported by caregivers and youth; and (3) physician training requirements and clinical implementation support. Priorities were ranked using consensus voting.

**Results:**

Top-ranked priorities varied by end-users. Caregivers prioritized pre-appointment information materials and stepwise instructions. Youth emphasized visual learning tools and app-based progress tracking. Physicians prioritized a streamlined script, needs assessment to guide training, and dissemination through conferences and in-person meetings. Cross-cutting themes included flexible delivery formats and ongoing implementation support beyond initial training.

**Conclusion:**

This consensus-building process with end-users identified distinct but complementary implementation priorities for Nova Sleepcare. These findings inform a multi-level implementation strategy addressing both clinical delivery and household adoption needs.

## Introduction

Up to 86% of children and youth with neurodevelopmental disorders (NDDs) have significant challenges with sleep quality and quantity, including difficulty falling asleep, frequent and/or prolonged overnight awakenings, and very early morning awakenings (1–3). Sleep problems contribute substantially to behaviors of concern (BoC; *e.g.,* aggression, self-injury) (4–7), with greater sleep disturbance associated with more severe behaviors and sleep improvements yielding behavioral improvements (8–10). At their most severe, BoC can prevent youth from safely living at home, limit future independence, and result in disproportionate healthcare utilization, estimated to be 2–5 times higher for children with severe BoC (11–18). The cascading effects of caregiver burnout, family stress, and reduced quality of life create an urgent need for accessible interventions (18–21).

Multiple factors are associated with sleep dysfunction experienced by youth with NDDs, including co-occurring medical and genetic conditions (*e.g.,* obstructive sleep apnea, chronic pain), environmental factors (*e.g*., noise and light pollution), lifestyle factors (*e.g.,* inadequate physical activity, screen use in bed), and alterations in the neurobiology of sleep (*e.g.,* altered or decreased release of the sleep hormone melatonin, circadian rhythm alterations) (10, 22–26). Behavioral insomnia is the most prevalent sleep problem among youth with NDDs (2). Recent evidence suggests that sleep problems in individuals with NDDs may begin in infancy (27–29), and emerging data show that single genes associated with some forms of NDDs play essential roles in regulating sleep (30–33).

Sleep education, the collaborative provision of sleep-related information to improve sleep, effectively addresses sleep problems in youth with NDDs regardless of underlying etiology (34, 35), serving as an essential first-line treatment without which other interventions often fail (36). Caregivers value this education and demonstrate strong improvements in youth sleep outcomes when able to access it with individualized support (37–42). Yet despite established efficacy and clinical guidelines (35), most children with NDDs never receive sleep interventions. A recent Canadian survey identified the following as critical barriers to receiving sleep interventions: specialized sleep clinics with months-long waitlists, multi-session programs incompatible with complex family schedules, and interventions lacking individualization for diverse presentations (43). This creates a significant mismatch: physicians identifying sleep problems during routine visits but lacking accessible interventions, sending families into queues for programs that may exclude youth with severe behaviors due to safety concerns. The result is a systematic failure to deliver evidence-based care when the need is identified. A recent literature review of pediatric behavioral sleep interventions underscored this crisis, calling for “access to scalable and easily disseminable tools” while noting “a lack of feasibility in implementing [current] programs in more accessible usual-care settings” (44).

To bridge this implementation gap, we developed and finalized the materials of Nova Sleepcare through iterative qualitative interviews with youth with NDDs, caregivers, and physicians (72). Unlike traditional sleep education programs, Nova Sleepcare embeds evidence-based content directly into routine pediatric appointments, delivered in a single visit by the diagnosing physician, individualized to each youth's diagnostic complexity and family's circumstances, and supported by materials for sustained stepwise implementation at home. This point-of-care model eliminates waitlists, leverages existing clinical relationships, and provides immediate support when problems are identified, while operating within standard pediatric clinical operations and their existing funding models rather than requiring separate infrastructure, specialized clinics, or additional funding streams that limit scale and sustainability.

However, developing effective content co-designed with end-users represents only part of the solution. Implementation science demonstrates that even evidence-based interventions fail without careful attention to delivery mechanisms, particularly for point-of-care models that must integrate into time-constrained clinical workflows (44–47). Successful implementation requires addressing physician training needs, creating efficient delivery tools, and ensuring families can translate in-clinic education to home practice (48–51). Critically, these implementation strategies must reflect end-user preferences rather than researcher assumptions to achieve sustainable adoption (45, 52).

Having established Nova Sleepcare's content through iterative qualitative interviews (72), we now address the equally critical question of implementation. We employed nominal group technique (NGT), a structured consensus method, to identify end-user priorities for learning about, implementing, and supporting Nova Sleepcare across clinical and household settings. This approach more closely ensures that implementation strategies align with the real-world needs of those who will deliver and receive the intervention.

## Methods

### Ethics statement

Institutional and operational approval (REB22-1525) was granted from the University of Calgary's Research Ethics Board and Alberta Health Services. An Advisory Council comprised of two youth with NDDs and two caregivers provided guidance throughout the research process, ensuring authentic research partner representation in study design, conduct, and analysis. Involvement varied depending on the specific Advisory Council member; at minimum, all members attended formal bi-monthly meetings, provided *ad hoc* feedback via email, and shared in decision making for this project. They were compensated financially for their time and essential contributions (see below for details). All Advisory Council members join this manuscript as co-authors (CH, GH, ED, SD).

### Nova Sleepcare

Nova Sleepcare is a point-of-care sleep education intervention delivered by physicians during routine clinical encounters with youth with NDDs experiencing BoC and sleep problems (72). The estimated time commitment per clinical encounter is ∼5–8 min. The intervention delivers active sleep education: a collaborative, physician-led process that engages families in developing individualized sleep strategies during clinical encounters, rather than passive information sharing. The intervention content, derived from evidence-based clinical trials and pediatric sleep guidelines (35, 36, 53, 54), was refined through qualitative interviews with youth with NDDs, caregivers, and pediatric physicians (72). Core components address sleep physiology, sleep hygiene, environmental modifications, cognitive-behavioral strategies, and evidence-based melatonin protocols. Implementation materials include a reference guide for physicians, youth and caregiver handouts, a goal-setting tool (“Pathway to Better Sleep”), and supplementary web resources (https://www.novasleepcare.ca), and was designed by a professional graphic design company (Barun Fox). Although information focusing on general insomnia-related symptoms informed Nova Sleepcare, this intervention is suitable for any child experiencing medical, behavioural, or other 24-hour sleep problems, including sleep apnea, restless leg syndrome, and sleep habits. Materials can be individualized during the clinical encounter to address specific youth and family needs and facilitate stepwise implementation beyond the initial appointment.

### Recruitment

We recruited participants through two pathways: clinical recruitment via the Owerko Centre at Alberta Children's Hospital, and community recruitment through the Alberta SPOR Support Unit's Patient Engagement Team, which distributed study information to sixteen national organizations. Youth participants (aged 8–17 years) had documented neurodevelopmental diagnoses with concurrent BoC and sleep problems or were siblings of youth meeting these criteria. Caregiver participants included biological, adoptive, foster parents, or grandparents of eligible youth. Pediatric physicians (general and subspecialist pediatricians) were recruited through professional networks. Compensation followed standard compensation guidelines: $25/hour for youth and caregivers per Canada's Strategy for Patient-Oriented Research, $30/hour for Advisory Council Members, and $100/hour for physicians per Canadian Medical Association guidelines. This study uses the term “research partners” to describe the “active collaboration between [youth, caregivers, and physicians] in designing solutions to a prespecified problem” (55).

### Data collection

We employed NGT, a structured consensus method ensuring equal participation among end-users through systematic idea generation, discussion, and priority ranking (56). Following established protocols with virtual adaptations, we conducted separate NGT sessions for each end-user group. Research partners received preparatory materials and focus questions one week before sessions so that they had time to consider their contributions to the topic, reach out privately for explanation or clarification, and ask for any additional accommodations (57). Sample size for each end-user group was determined based on the recommendation of no more than seven participants per group (58).

The implementation questions were tailored to each end-user group's unique perspective (Table 1). Sessions followed the modified NGT protocol: individual idea generation (5 min), round-robin idea sharing with digital documentation, group clarification and discussion, and anonymous priority ranking of top five items per question (57). Virtual sessions were facilitated by trained researchers using Google Jamboard, a digital collaboration tool for real-time documentation and voting. For the physician group, an asynchronous format was used to mitigate scheduling conflicts, and undue influence of professional hierarchies (*e.g.,* physician seniority).

**Table 1:** Nominal group technique (NGT) questions.

End-user group
Caregiver
How would you like to learn about Nova Sleepcare from the pediatric physician? What potential barriers do you perceive that may hinder the implementation of Nova Sleepcare in your household?
Youth
How would you like to learn about Nova Sleepcare from the pediatric physician? How could we help youth like you use Nova Sleepcare at home to successfully improve sleep?
Physician
How could we best train pediatric physicians to deliver Nova Sleepcare? How could the implementation of Nova Sleepcare be supported in different clinical settings?

### Data analysis

Demographic characteristics of research partners were summarized using descriptive statistics, with frequencies and percentages reported for categorical variables.

For the NGT data, priorities were analyzed following established NGT protocols (56–58), Each participant ranked their five highest priorities per question, assigning scores from 5 (highest priority) to 1 (lowest priority). Sum scores were calculated by aggregating rankings across participants within each group. Items were then ordered by sum score to identify group priorities, with higher sum scores indicating greater importance. When items received identical sum scores, they were differentiated by voting frequency (number of participants who included the item in their rankings). This approach yielded ranked priorities for each question within each end-user group, facilitating comparison of implementation needs across caregivers, youth, and physicians. The NGT priorities were presented, reviewed, and finalized by the Advisory Council during a separate research activity; they did not directly participate in the NGT process to avoid duplicate sampling.

## Results

### Demographics

Table 2 presents the demographic characteristics of the 15 end-users who participated in the nominal group technique sessions. The caregiver group (*n* = 6) consisted of biological mothers aged 35–49 years, with most being married or in common-law relationships (*n* = 5). This group demonstrated socioeconomic diversity across the income spectrum, with participants reporting household incomes from under $60,000 to over $200,000, and educational attainment spanning from diplomas to graduate degrees. All caregivers resided in large urban centers, with two-thirds being Canadian citizens by birth.

**Table 2:** Demographics of End-User groups.

Demographic variables	Caregivers	Youth	Physicians
	*n* = 6 (%)	*n* = 4 (%)	*n* = 5 (%)
Age			
8–10	–	1 (25%)	–
11–13	–	3 (75%)	–
30–34	–	–	2 (40%)
35–39	3 (50%)	–	1 (20%)
40–44	2 (33%)	–	–
45–49	1 (17%)	–	2 (40%)
Sex			
Female	6 (100%)	1 (25%)	3 (60%)
Male	–	3 (75%)	2 (40%)
Gender			
Man or Boy	–	3 (75%)	2 (40%)
Woman or Girl	–	1 (25%)	3 (60%)
Race			
White	3 (50%)	2 (50%)	3 (60%)
South Asian	2 (33%)	–	–
East Asian	–	–	2 (40%)
Latino	1 (17%)	1 (25%)	–
No Response	–	1 (25%)	–
Immigration Status			
Canadian citizen by birth	4 (67%)	4 (100%)	5 (100%)
Canadian citizen by naturalization	2 (33%)	–	–
Relationship to Youth Partner			
Biological Mother	6 (100%)	–	–
Relationship Status			
Married or Common-Law	5 (83%)	–	–
Divorced or Separated	1 (17%)	–	–
Community of Residence			
Large urban population, with a population of 100,000 or more	6 (100%)	–	–
Household Income			
$30,000–$59,000	1 (17%)	–	–
$100,000–$149,000	2 (33%)	**–**	**–**
$200,000 and over	2 (33%)	**–**	**–**
No Response	1 (17%)		
Current Employment Status			
Part Time	2 (33%)	**–**	**–**
Full Time	3 (50%)	**–**	**–**
Other	1 (17%)	**–**	**–**
Highest Level of Education			
Undergraduate Degree/Bachelor's Degree	2 (33%)	**–**	**–**
Graduate Degree	2 (33%)	**–**	**–**
Diploma or Certificate	2 (33%)	**–**	**–**
NDD Diagnosis^a^			
Autism Spectrum Disorder	**–**	1 (25%)	**–**
Attention Deficit Hyperactivity Disorder	**–**	3 (75%)	**–**
Specific Learning Disorder	**–**	2 (50%)	**–**
Co-occurring Condition			
Dysgraphia and Dyscalcula	**–**	1 (25%)	**–**
Years of Practicing Medicine			
3	**–**	**–**	1 (20%)
5	**–**	**–**	1 (20%)
7	**–**	**–**	1 (20%)
14	**–**	**–**	1 (20%)
16	**–**	**–**	1 (20%)
Location of Practice			
Large urban population, with a population of 100,000 or more	**–**	**–**	5 (100%)
Practice Setting			
Private Practice	**–**	**–**	2 (40%)
Hospital	**–**	**–**	1 (20%)
Combination	**–**	**–**	2 (40%)
Specialty			
Developmental Pediatrician	**–**	**–**	1 (20%)
General Pediatrician	**–**	**–**	3 (60%)
Neurologist	**-**	**-**	1 (20%)

a Sums will not equal to 100.

The youth participants (*n* = 4) ranged from 8 to 13 years of age, and most were males (*n* = 3). All youth experienced sleep problems and were diagnosed with at least one neurodevelopmental disorder, most commonly attention-deficit/hyperactivity disorder (*n* = 3), followed by specific learning disorders (*n* = 2) and autism spectrum disorder (*n* = 1). All youth participants were Canadian citizens by birth.

The physician group (*n* = 5) included three general pediatricians, one developmental pediatrician, and one pediatric neurologist, with clinical experience ranging from 3 to 16 years. All physicians practiced in large urban settings with diverse practice environments including private clinics (*n* = 2), hospitals (*n* = 1), and combination settings (*n* = 2). The physician group comprised three female and two male physicians, with representation from both Canadian-born and naturalized citizens.

#### End-user priorities for learning about Nova sleepcare

Caregivers and youth demonstrated convergent and divergent priorities for learning about Nova Sleepcare (Table 3). Caregivers' highest priority (sum score = 14) was receiving materials before appointments to review and prepare questions. They also prioritized screening for sleep apnea (sum score = 12) and concise, in-visit explanations of behavioral sleep strategies combined with visual materials (sum score = 9). Youth strongly favored video-based learning, ranking in-clinic videos as their top priority (sum score = 14), followed by pre-appointment videos (sum score = 10). Both groups valued technology-enhanced approaches, with caregivers prioritizing a digital platform (*e.g.,* confidential family app) to navigate sleep goals and challenges (sum score = 8) and youth ranking an informational app third (sum score = 8). While specific learning preferences differed, both caregivers and youth independently prioritized in-person, collaborative engagement during clinical visits: caregivers through joint goal setting (sum score = 7) and youth through direct physician dialogue (sum score = 7), underscoring the importance of shared decision-making with both caregivers and youth in Nova Sleepcare's implementation model.

**Table 3:** Learning priorities for Nova Sleepcare.

**Caregiver Learning Priorities**	**Score**	**Rank**	**Youth Learning Priorities**	**Score**	**Rank**
Pre-appointment materials for review	14	1	In-clinic video demonstration	14	1
Screening for sleep apnea	12	2	Pre-appointment video	10	2
Brief explanation with visual aids	9	3	Information app with videos	8	3
Technology-enhanced family app	8	4	In-clinic, physician Q&A	7	4
Joint goal-setting visit	7	5	Bedside visual reminder poster	7	5

#### Household implementation barriers and support needs

Caregivers identified scheduling complexity as the primary barrier to implementing consistent routines to support sleep (sum score = 17), substantially outranking other concerns (Table 4). Three barriers tied for second priority (sum scores = 10 each): inconsistent implementation of routine sleep supports between caregivers, late afterschool activities disrupting bedtime, and difficulty maintaining routines during and after vacation upon return. Finally, caregivers with large families cited complex schedules as a barrier to consistent routines. Youth prioritized understanding Nova Sleepcare's benefits (sum score = 14), visual cues (*e.g.,* poster) for reminders of sleep supports (sum score = 12), bedside checklists to track progress towards implementing sleep supports (sum score = 6), and a digital platform (*e.g.,* app) with rewards for progress achievements (sum score = 8) (Table 4).

**Table 4:** Household implementation barriers and support strategies.

**Caregiver Identified Barriers**	**Score**	**Rank**	**Youth Identified Support Aids**	**Score**	**Rank**
Complex/changing schedules	17	1	Understanding Nova Sleepcare's benefits	14	1
Cross caregiver consistency	10	2	Poster with sleep hygiene steps	12	2
Late afterschool activities	10	3	Progress tracking website with rewards	8	3
Vacation schedule disruption	10	4	All-in-one Nova Sleepcare app	8	4
Large family dynamics	7	5	Bedside tracker/checklist	6	5

#### Physician training and clinical implementation priorities

Physicians identified Nova Sleepcare delivery materials that were streamlined as their top training priority (sum score = 12; Table 5). Conference presentations of Nova Sleepcare ranked second (sum score = 11), followed by needs assessments to tailor training approaches (sum score = 10) due to differences in physician familiarity with the material and experience in managing sleep problems. Physicians also valued in-person training sessions and requested comparisons with existing sleep programs to demonstrate Nova Sleepcare's advantages and facilitate buy-in of the program. For clinical implementation support (Table 5), printed materials dominated as the highest priority (sum score = 19), substantially exceeding other supports. Educational strategies emphasizing key concepts (*e.g.,* sleep physiology, melatonin, sleep strategies) ranked second (sum score = 13), while expanding training to non-pediatric providers, gathering implementation feedback, and developing digital versions each received equal priority (sum scores = 9).

**Table 5:** Physician identified implementation priorities.

**Training Priorities**	**Score**	**Rank**	**Clinical Support Priorities**	**Score**	**Rank**
Streamlined visual reference guide	12	1	Printed materials (colored)	19	1
Conference presentations	11	2	Educational strategies (teach 3–5 key concepts)	13	2
Needs assessment for physician training	10	3	Non-pediatric provider training	9	3
In-person training	9	4	Implementation feedback system	9	4
Comparison to alternative sleep education programs for buy-in	8	5	Electronic/app versions of Nova Sleepcare	9	5

#### Cross-Cutting implementation themes

Analysis across all end-user groups revealed consistent themes: preference for multimodal learning materials (visual, digital, print), the need for both pre-visit preparation and in-visit support, and tools that facilitated sustained implementation beyond initial encounters. These convergent priorities suggest a multi-level implementation strategy addressing both immediate clinical needs and long-term household adoption.

To facilitate implementation trial planning, we mapped these priorities to established implementation outcomes (59). Knowledge transfer strategies dominated across all groups (11 of top priorities). There were fewer sustainability strategies prioritized (4 priorities), which is an important finding for future implementation planning. Complete mapping of these priorities appears in Supplementary Tables S1–S4.

## Discussion

The purpose of the current study was to use consensus methods to understand how end-users (neurodiverse youth, their caregivers, and pediatric physicians) prioritize learning about, implementing, and supporting Nova Sleepcare across diverse clinical and household contexts. Our findings revealed both convergent themes and group-specific needs across youth, caregivers, and physicians that inform a comprehensive implementation strategy. Across all end-user groups, three unifying priorities emerged: the critical importance of pre-appointment preparation, the need for multimodal learning materials, and the emphasis on tools that support stepwise and sustained implementation beyond initial clinical encounters. While these convergent themes provide a foundation for implementation, important divergent priorities reflect each group's unique perspective and constraints. Caregivers focused on the practical challenge of managing complex family schedules—a barrier that substantially outweighed all other implementation concerns—while youth prioritized understanding why Nova Sleepcare would help them and having engaging visual reminders in their environment. Physicians prioritized streamlined, visually-oriented delivery tools that could integrate efficiently into time-constrained clinical workflows, coupled with comprehensive training that acknowledges varying levels of sleep management expertise.

These findings fulfill our objective of identifying implementation priorities through structured consensus methods, providing a roadmap for translating Nova Sleepcare from a co-designed intervention into sustainable clinical practice. Specifically, these priorities (*e.g.,* pre-appointment videos) can also be translated to key features of Nova Sleepcare (*e.g.,* in-app sleep diary) for the next phase of study (Table 6). For example, posters and visual reminders are highly actionable priorities, and will be achieved through access to materials via the Nova Sleepcare website and a mobile application (in development) for youth and caregivers. Materials can be accessed pre-appointment and the use of sleep strategies after the in-clinic delivery of Nova Sleepcare can be tracked. The use and type of Nova Sleepcare materials in clinic is dependent on the discretion of physicians (*e.g.,* print vs. digital materials), which allows the physician to customize delivery to best meet the needs of their patients and their own clinical workflow. Youth and caregivers can also access these materials after the appointment to help support sleep-problem solving for different household environments. Importantly, the delineation between universal implementation needs and group-specific requirements suggests that successful implementation must be both standardized in its core components and flexible in addressing the distinct challenges faced by individual families and clinicians.

**Table 6:** A traceability matrix for NGT priorities.

**NGT Priorities**	**Specific Intervention Features**
Pre-Appointment Materials	Website or App (in development)
In-clinic video demonstration	Website or In-App Content Section
Pre-appointment video	Website or In-App Content Section
Brief explanation with visual aids	Clinical appointment, In-App Content Section
Information app with videos	In-App Content Section
Technology-enhanced family app	App (in development)
In-clinic, physician Q&A	Clinical appointment
Joint goal-setting visit	Clinical appointment
Bedside visual reminder poster	In-App Sleep Diary

Our implementation findings validate and extend the co-design priorities that shaped Nova Sleepcare's development (72). Our companion study revealed that end-users had limited personal resources and capacity to implement sleep strategies, with caregivers describing feeling overwhelmed by too much information and physicians requesting streamlined, stepwise approaches. In direct response, we developed the “Pathway to Better Sleep”—a priority goal-setting framework that delivers content in manageable, individualized steps. This component of Nova Sleepcare is now validated by our implementation findings, where caregivers' primary barrier was managing complex household schedules (sum score = 17), and their highest learning priority was receiving materials for pre-appointment review and preparation (sum score = 14). The Pathway to Better Sleep framework directly addresses both needs by breaking behavioural sleep education into discrete, achievable goals that youth, caregivers, and physicians can jointly select based on youth diagnostic complexity, family capacity, and current circumstances. Although behavioral change can be difficult to achieve, as this requires long-term commitment and consistency from end-users (50), using strategies to improve sleep can have an impact the first night they are implemented, positively impacting children and families as soon as the following day. This has the potential to positively support behavioural change around sleep.

Critically, Nova Sleepcare's emphasis on shared decision-making—identified as essential during co-design—emerged as a convergent implementation priority. While caregivers and youth expressed different learning preferences in this study, both groups independently prioritized collaborative, in-person engagement during clinical visits. This alignment is particularly significant given our co-design finding that youth and caregivers often held divergent views on sleep problems and solutions. Youth in the companion study articulated sophisticated understanding of how sleep affected their emotional and social functioning, yet traditional sleep programs often excluded them from clinical discussions, teaching only caregivers who then instructed youth regarding changes at home. Our implementation data reinforces this gap: youth prioritized understanding the benefits of Nova Sleepcare (sum score = 14), having direct dialogue with physicians (sum score = 7), and using environmental cues they could control (sum score = 12). The physician's role as trusted mediator, central to Nova Sleepcare's co-design, addresses this implementation challenge. As established in the companion paper, physicians requested tools that could guide rather than prescribe, enabling them to mediate compromises between youth and caregiver perspectives. This collaborative approach aligns with evidence that physician-mediated goal setting improves adherence across complex health conditions (60–63). A potential tension was observed between youth autonomy and caregiver compliance towards the priorities set forward, where youth emphasized for visual and environmental supports, whereas caregivers focused on consistent schedules and routines. Nova Sleepcare provides materials that can support both youth and caregiver needs. By positioning physicians and caregivers as collaborative partners with youth, Nova Sleepcare facilitates youth engagement in their own behavior change: a critical contrast to top-down models that can provoke resistance and undermine sustained adherence.

Integrating these priority areas as part of Nova Sleepcare directly address barriers identified in recent implementation research. Several studies found that families using self-directed sleep programs report being overwhelmed by excessive information that often poorly aligns with their child's specific needs (64–66). Similarly, participants in online sleep education programs cite information overload and lack of implementation support as primary reasons for low engagement and failure to complete the program (67). Nova Sleepcare's co-design process anticipated these barriers: iterative refinement with end-users resulted in content described as accessible, comprehensive yet digestible, and, crucially, deliverable within routine clinical encounters where individualization occurs naturally through physician-family dialogue. Additionally, our results have motivated development of a Nova Sleepcare mobile application (currently underway) in order to integrate youth perspectives towards high-engagement videos and visual cues as necessary supports for sleep-problem solving. This will support sustainability and sustained behaviour change for end-users.

Pediatric physicians in our study highlighted the need for multiple training opportunities and for Nova Sleepcare to be offered in multiple formats, including printed materials and a mobile platform for easy access. We will use these suggestions in the next phase of our work, in which we will train physicians to deliver Nova Sleepcare and test the feasibility of in-clinic delivery. An important consideration is the time required, given the realities of the time demands of being a physician. We will assess the time commitment and preferred format of training within real clinical workflows—importantly, we will receive ongoing physician feedback regarding the training to determine the minimum training required to maintain fidelity.

Unlike traditional sleep education programs, Nova Sleepcare embeds evidence-based content directly into routine pediatric encounters. This point-of-care model eliminates waitlists, leverages existing clinical relationships, and provides immediate support when problems are identified, rather than requiring separate infrastructure, specialized clinics, or additional funding streams that limit scale and sustainability. This model also responds to the “implementation cliff,” the sharp drop in intervention benefit when moving from controlled research environments into real-world practice (68). By aligning delivery with routine care workflows and using practitioners already embedded in the care system, Nova Sleepcare preserves intervention fidelity while ensuring pragmatic feasibility. In contrast to externally funded programs that may falter when grants expire or require specialized staff and new operations infrastructure, Nova Sleepcare's point-of-care model can be scaled without establishing a parallel stream of clinical activity.

Importantly, Nova Sleepcare can address known limitations in the field of behavioral sleep interventions (BSI), including a lack of adaptations to NDD-specific needs and co-occurring conditions (*e.g.,* chronic medical conditions, behavioral concerns) (69), and challenges with accessing or receiving effective behavioral sleep interventions (70). For example delivering print materials in clinics where pediatric physicians have regular contact with patients and sleep strategies that end-users can use immediately without requiring further training or expertise can address access, timeliness, and socio-demographic barriers faced by many families (43, 44). In response to skewed sampling of participants identified as white with moderate-to-high educational levels in research focusing on BSI, and the growing emphasis to recruit children and caregivers of ethnic minority backgrounds (71), we undertook purposeful sampling to ensure diversity across diagnosis type, developmental age groups, and socio-demographic and socio-economic factors (Table 2). However, we acknowledge that further work is required to ensure Nova Sleepcare is suitable for all families regardless of socio-demographic and socio-economic factors.

As a next step, Nova Sleepcare will be tested in a formal implementation trial. Pre-defined metrics for implementation success include high acceptability, appropriateness, and feasibility ratings across clinical delivery and patient uptake, fidelity scores, and positive improvements in sleep and behavioral outcomes. This phase will prioritize purposeful sampling across diverse patient populations to find ways to make clinical delivery and at-home uptake easier in resource limited settings, including the use of materials that require low digital literacy.

## Limitations

Limitations of this study include a geographically limited sample and English-only interviews, which may reduce generalizability and influence the priorities identified. To address this in future phases, Nova Sleepcare materials have been translated into French and Spanish to broaden reach across non-English speaking households; translation into additional languages will soon follow. While our sample size was small, there is a lack of existing guidelines to determine sample size for consensus-methods, like NGT (56). We did not conduct further sessions because a reasonable list of priorities was generated, and complemented insights in the companion paper. Moreover, a formal implementation trial will generate further insights toward physician training, clinical delivery, and patient uptake. Finally, the use of a virtual format, while practical, may have constrained opportunities for spontaneous elaboration or clarification, potentially leaving some ideas underexplored.

## Conclusion

This study underscores that co-design must extend beyond intervention content to include delivery models that reflect real-world constraints and end-user priorities and capacities. Nova Sleepcare offers a pragmatic blueprint for point-of-care translation, embedding evidence-based sleep education into routine clinical workflows without requiring additional infrastructure, specialized staffing, or parallel funding streams. As implementation planning becomes recognized as essential to intervention development, this approach highlights the value of designing for scale and sustainability from the outset. Building on these findings, future research should compare economic implications of different delivery models (*e.g.,* virtual, allied health), and assess long-term sustainability in diverse pediatric care settings. These next steps will help advance a broader shift toward implementation-ready design in pediatric sleep interventions and behavioral health.

## Data Availability

The original contributions presented in the study are included in the article/Supplementary Material, further inquiries can be directed to the corresponding author.
