# Supplementary material for: Co-designing Nova Sleepcare: identifying priority areas for implementation
Source: Front Pediatr. 2026 Jul 1;14:1760214. doi: 10.3389/fped.2026.1760214 (PMC13368799; doi:10.3389/fped.2026.1760214)
Supplement: Supplementary file 1 [file Table1.docx]

**Table S1: Knowledge Transfer Strategies (How Information is Shared to Support Uptake)**

| **Strategy** | **End-User Group** | **Priority Score** | **Rank** |
| --- | --- | --- | --- |
| Printed versions available (colored handouts) | Physicians | 19 | 1 |
| Email/send materials for review and question preparation | Caregivers | 14 | 1 |
| Video about Nova Sleepcare during clinic visit | Youth | 14 | 1 |
| Explain to youth what Nova Sleepcare does so they know it will help | Youth | 14 | 1 |
| Employ teaching strategies (3-5 key concepts) | Physicians | 13 | 2 |
| Screening for sleep apnea | Caregivers | 12 | 2 |
| Streamlined visual reference guide (not script) | Physicians | 12 | 1 |
| Present at multiple conferences | Physicians | 11 | 2 |
| Watch video about Nova prior to appointment | Youth | 10 | 2 |
| Conduct needs assessment for training | Physicians | 10 | 3 |
| Brief key points combined with poster/handout visuals | Caregivers | 9 | 3 |

**Table S2: Engagement Strategies (What Promotes Adoption)**

| **Strategy** | **End-User Group** | **Priority Score** | **Rank** |
| --- | --- | --- | --- |
| Glow-in-the-dark poster on ceiling with steps | Youth | 12 | 2 |
| In-person training meetings | Physicians | 9 | 4 |
| Confidential app for families to navigate goals | Caregivers | 8 | 4 |
| Website to track progress and get rewards | Youth | 8 | 3 |
| App with everything about Nova in one place | Youth | 8 | 4 |
| Give physicians reasons to use Nova over alternatives | Physicians | 8 | 5 |
| Joint visit with goal setting and check-ins | Caregivers | 7 | 5 |
| Ask doctor questions in person | Youth | 7 | 4 |
| Poster with steps for bedside | Youth | 7 | 5 |

**Table S3: Sustainability Strategies (What Maintains Use)**

| **Strategy** | **End-User Group** | **Priority Score** | **Rank** |
| --- | --- | --- | --- |
| Training non-pediatric providers | Physicians | 9 | 3 |
| Seeking feedback after implementation period | Physicians | 9 | 4 |
| Electronic/mobile app versions | Physicians | 9 | 5 |
| Poster with tracker/checklist | Physicians | 6 | 5 |

**Table S4: Barriers Identified (What Inhibits Adoption)**

| **Barrier** | **End-User Group** | **Priority Score** | **Rank** |
| --- | --- | --- | --- |
| Managing complex/busy schedules | Caregivers | 17 | 1 |
| Consistency across caregivers | Caregivers | 10 | 2 |
| Late afterschool activities affecting bedtime | Caregivers | 10 | 3 |
| Maintaining schedules on vacation | Caregivers | 10 | 4 |
| Large families managing multiple schedules | Caregivers | 7 | 5 |
